# Study and Modelling of Fluid Flow in Ceramic Foam Filters

**DOI:** 10.3390/ma16175954

**Published:** 2023-08-30

**Authors:** Massoud Hassanabadi, Shahid Akhtar, Ragnhild E. Aune

**Affiliations:** 1 Department of Materials Science and Engineering, Norwegian University of Science and Technology (NTNU), 7491 Trondheim, Norway; ragnhild.aune@ntnu.no; 2 Hydro Aluminium, Karmøy Primary Production, Håvik, 4265 Karmøy, Norway; shahid.akhtar@hydro.com

**Keywords:** ceramic foam filters (CFFs), permeability of CFFs, COMSOL Multiphysics^®^, aluminium filtration, DC casting of aluminium, aluminium recycling

## Abstract

To investigate the fluid flow characteristics of conventional Ceramic Foam Filters (CFFs) of grades 30 and 50, a 2D macro-scale geometry was generated by converting pixel grid images of the filters into vector format images. The flow behaviour through the filter channels was then numerically modelled using the Stocks equation within the Creeping Flow interface of COMSOL Multiphysics^®^. Through modelling, the average interstitial velocity was estimated and found to be higher than the corresponding value obtained from the Dupuit–Forchheimer equation. The discrepancy obtained suggested that the flow behaviour within the filter channels differed from that based on the simplified assumptions of the equation. The porosity and permeability of the CFFs were evaluated during the post-processing stage using surface integration and user-defined equations. The experimentally determined porosity closely matched the values obtained from the simulation model, demonstrating the reliability of the numerical approach. However, the permeability values from the simulation of CFFs of grades 30 and 50 were higher than those obtained experimentally. This discrepancy can be attributed to the larger channels in the generated geometrical pattern compared to the original CFF structure. The present findings highlight the effectiveness of the proposed methodology in developing a representative macro-scale geometry for CFFs and in simulating fluid flow behaviour.

## 1. Introduction

Non-metallic inclusions introduced during the melting, alloying, and transfer processes in aluminium production are effectively removed before casting by employing alumina (Al_2_O_3_)-based Ceramic Foam Filters (CFFs) as a final refining step [1]. The primary mechanisms involved in particle removal within CFFs are interception and gravitation [2]. Interception occurs when the edge of small particles, with a density lower than that of molten aluminium, comes into contact with the surface of the collector, i.e., of the filter *Strut*, as the particles follow the streamlines. If the interfacial tension between a particle and the collector is lower than the interfacial tension between the particle and the melt, as well as than that between the melt and the collector, the particles are captured by the collector [3,4]. In the gravitation mechanism, the particles with higher density than that of the molten aluminium settle out in the direction of the gravitational forces [5]. The combined effects of interception and gravitation collisions are typically used to assess the filtration efficiency of CFFs [3,5]. Additionally, the filtration efficiency is influenced by the fluid flow characteristics, which play a significant role in determining the transport of inclusions to the filter collectors and the re-entrainment of captured inclusions [6].

Aluminium filtration typically occurs at a velocity of approximately 10 mm/s [1,7], and the corresponding flow within the CFF pores is considered to follow a creeping flow regime, where the interstitial Reynolds number (*Re_i_*) is less than unity [3]. Understanding the flow through CFFs is crucial for comprehending various flow regimes, dominant forces (such as viscous and drag forces), and the associated pressure drops and permeabilities [8]. Additionally, the process involves utilising a metallic static head to initiate the priming of the filters. This action displaces the air within the tortuous structure of the filters, facilitating the subsequent infiltration of the porous material [1,9,10]. The magnitude of the metallic static head is contingent upon the permeability of CFFs, a critical factor that significantly influences the design of the filter box for the filtration of aluminium in the direct-chill casting process.

The permeability of Al_2_O_3_-based CFFs was empirically evaluated using water as a surrogate due to its similar dynamic viscosity to that of molten aluminium at the casting temperature, i.e., 993–1003 K [9,10,11,12]. As water permeates through the CFF, the permeability is determined by correlating the measured pressure gradient to the superficial velocity using the Darcy equation within the range of the creeping flow regime, according to Equation (1) [13]:

(1) $$-\nabla P=\frac{\mu }{k}u$$

where ∇P is the pressure gradient (kg/m^2^∙s^2^ or Pa/m), μ is the dynamic viscosity (kg/m∙s), k is the intrinsic permeability of the porous medium (m^2^), and ***u*** is the superficial (seepage) velocity (m/s), which is the flow velocity expressed as the ratio of the volumetric flow rate to the cross-sectional area from which the fluid is flowing.

Numerical simulations provide an alternative approach to conventional empirical methods because conducting experiments is often expensive and time-consuming. In view of this, the study of fluid flow through porous media has, over the years, been performed using various approaches, including pore-scale generation methodologies and numerical models for computing flow fields within the complex void space of the filter [14,15,16,17,18].

Regulski et al. [15] numerically investigated the transport properties in 3D models of ceramic foams reconstructed from Computer Tomography (CT) scans. They utilised the Lattice–Boltzmann Method (LBM), an alternative to the conventional Computational Fluid Dynamics (CFD) methods like the Finite Element Method (FEM) and the Finite Volume Method (FVM). Their results exhibited excellent agreement with the experimentally obtained pressure drop data for CFFs with 10 and 20 Pores Per Inch (PPI). However, the 30 PPI filters showed less satisfactory agreement due to the smaller and heterogeneous volume of the CT image, in this case.

Similarly, Carvalho et al. [16] performed a pore-level numerical simulation on a 3D digital representation of a metallic foam generated from CT scans. Their results indicated that the numerically determined permeability was one order of magnitude lower than the corresponding experimental value. This discrepancy was attributed to the effect of the surface roughness of the foam, which was assumed to be completely smooth in the model. Another study by Westhoff et al. [17] investigated the permeability of open-cell foams generated using 3D stochastic models. The permeability values were validated experimentally through pressure drop tests after 3D-printing the structures. Even another study by Petrasch et al. [18] focused on determining the permeability and *form drag coefficients* by simulating the transport properties of flow in a 3D digital representation of a 10 PPI reticulated porous ceramic. The permeability obtained from the numerical simulation was compared to the values predicted by analytical equations, with the two-point correlation bound [19,20], providing the most accurate estimation in line with the simulation data.

In the present work, 2D geometrical patterns of Al_2_O_3_-based CFFs with grades 30 and 50 were generated, and numerical CFD simulations of a water flow through the channels was performed using COMSOL Multiphysics^®^ as the platform for solving the CFD codes. The modelling results were utilised for postprocessing the numerical data to evaluate the permeability of the CFFs.

## 2. Experimental Procedure and Numerical CFD Model

**Figure 1:**
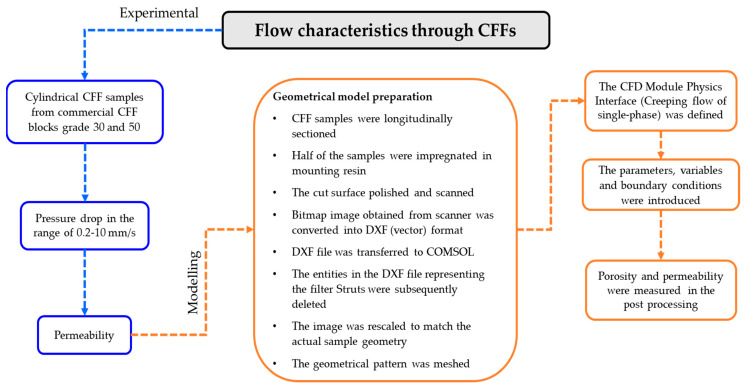
Experimental and CFD modelling procedures adopted to determine the porosity and permeability of CFFs of grades 30 and 50.

The flowchart presented in Figure 1 shows the step-by-step process adopted in both the experimental and the modelling phases of this work. Further details of the overall procedure are provided in subsequent sections for a comprehensive understanding.

### 2.1. Material and Generation of a Macro-Structural Model

**Figure 2:**
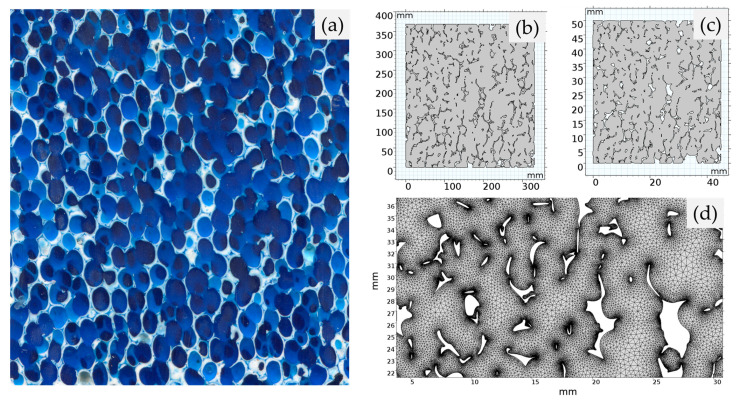
The procedure for generating a 2D geometrical model of CFFs for CFD modelling using COMSOL Multiphysics^®^. (**a**) Scanning the impregnated CFFs with coloured metallographic castable mounting resin. (**b**) Conversion of the scanned image type from bitmap to DXF. (**c**) Removal of the region representing the filter *Struts* in the DXF file. (**d**) Meshing.

Commercial CFFs of grades 30 and 50, measuring 584.2 × 584.2 × 50.8 mm, were used to create cylindrical samples. The filter samples were longitudinally sectioned, and half of the sections were impregnated with blue metallographic castable mounting resin using a vacuum mounting apparatus. This impregnation process enhanced the resin penetration into the voids and facilitated the release of any trapped bubbles. The surface of the sectioned sample, parallel to the height, was ground to remove excess resin and then polished to eliminate scratches and, thereby, obtain a smooth surface. A flatbed scanner (Perfection V330 Photo Scanner from Seiko Epson Corporation, Tokyo, Japan) with a resolution of 2000 dpi was used to scan the sample surface. The scanned images were processed to recover the geometry pattern of the CFFs for importation into the simulation software, i.e., into COMSOL Multiphysics^®^ (version 5.3). Figure 2a–d illustrates the steps involved in preparing the geometric pattern. Figure 2a shows the scanned image of the CFF samples impregnated with castable resin, where the white filaments represent the filter *Struts*, and the blue regions represent the channels. By converting the scanned images into a vector format, distinct divisions within the image were created due to the colour contrast between the resin and the CFF material. In view of this, the bitmap images obtained from optical scanning were converted to the Drawing Exchange Format (DXF), as shown in Figure 2b, and then imported into the simulation software. The entities in the DXF file representing the filter *Struts* were subsequently deleted using the functionalities in the Model Geometry interface, resulting in Figure 2c. The image was then rescaled to match the actual sample geometry. Finally, as shown in Figure 2d, the models were meshed using the physics-controlled mesh sequence type with a standard element size.

### 2.2. Parameters, Variables and Governing Equations

Table 1 summarises the fluid properties, the boundary condition, and the geometric dimensions introduced into the numerical CFD model as inputs. The pressure values listed in Table 1 are initial values, and a *Parametric Sweep* study was performed with a sequence of pressure drops within the region of interest.

The *Volumetric flow rate through the outlet*, the *Darcy velocity at the outlet* and the *Permeability* were defined as user-defined variables for post-processing evaluation of the permeability. Table 2 presents the user-defined variables along with their expressions and units.

### 2.3. Boundary Conditions and Assumptions

**Figure 3:**
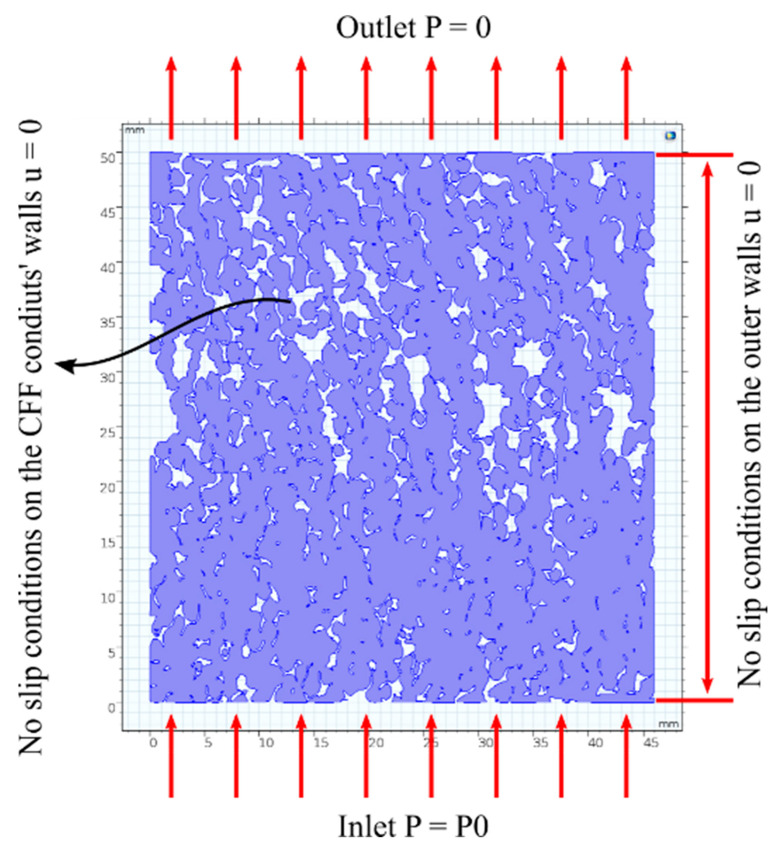
Computational domain with boundary conditions for the geometric pattern of the CFFs of grade 50.

Figure 3 illustrates the geometric pattern of CFFs of grade 50 and the corresponding boundary conditions. The same boundary conditions were also applied to the numerical CFD model for CFFs of grade 30.

A uniform pressure drop at the inlet was assumed, and the COMSOL Multiphysics^®^ (version 5.3) CFD code was used to solve the incompressible continuity and Stokes equations in the pore space geometry of the CFFs.

(2) $$\nabla P=\mu \nabla^{2}u+F$$

(3) $$\frac{D\rho }{Dt}=-\rho \nabla .u$$

where *P* is the pressure force (Pa), *µ* is the dynamic viscosity (kg/m∙s), ***u*** is the velocity vector (m/s), *F* indicates all forces other than the pressure, and *ρ* is the fluid density (kg/m^3^). Equations (4) and (5) involve the *Laplace operator* and the *substantial time derivative* ($\frac{D\rho }{Dt}$), respectively.

(4) $$\nabla^{2}=\Delta =\frac{\partial^{2}}{\partial x^{2}}+\frac{\partial^{2}}{\partial y^{2}}+\frac{\partial^{2}}{\partial z^{2}}$$

(5) $$\frac{D\rho }{Dt}=\frac{\partial \rho }{\partial t}+u_{x}\frac{\partial \rho }{\partial x}+u_{y}\frac{\partial \rho }{\partial y}+u_{z}\frac{\partial \rho }{\partial z}$$

The Stokes equation is a simplified version of the Navier–Stokes equation and is applicable when *Re_i_* is less than 1. By neglecting the time dependence and advective terms, the computational analysis of the Stokes equation is less memory-intensive and tedious compared to the full Navier–Stokes equation. Consequently, the experimental pressure drops corresponding to the velocity data points that provided *Re_i_* numbers that were less than 1 were implemented as a *Parametric Sweep* in the model. Table 3 summarises the boundary conditions, while Table 4 outlines the assumptions made in the analysis.

### 2.4. Experimental

The hydraulic properties of CFFs of grades 30 and 50 were experimentally investigated through pressure drop experiments performed within a velocity range of 0.2–10 mm/s. A dedicated experimental set-up was designed and constructed for the purpose (a detailed description of the set-up was published elseward [21]).

**Figure 4:**
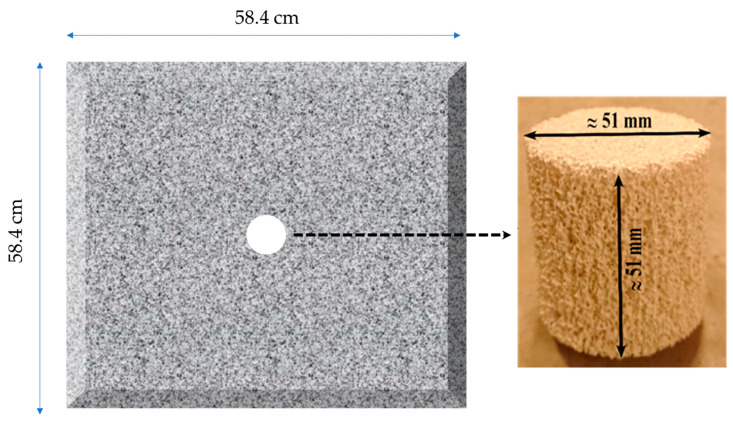
The location from which the sample was extracted within a CFF tile/block. The secured sample on the right-hand side of the image corresponds to a CFF of grade 50.

The CFF samples selected for the investigation were obtained by cutting commercial alumina (Al_2_O_3_) CFF tiles/blocks measuring 58.4 × 58.4 × 5.1 cm^3^ using a core drill machine, resulting in samples with a nominal diameter of 51 mm. As shown in Figure 4, the samples were taken from the centre of the tiles/blocks. To ensure accurate measurements, the samples were subjected to a specific drying process after being cut, as they were exposed to water during the cutting process, i.e., heated to 423 K for 12 h. Once completely dried, the weight and dimensions of each sample were promptly measured using precise equipment, with multiple measurements recorded for each parameter.

In preparation for the experiments, the CFF samples were carefully mounted in the sample holder to minimise any potential bypassing effects caused by the exterior walls of the sample holder or by the surface of the sample. Also in this case, a detailed description of the preparation procedure for the CFF samples, the sample holder design, the test procedure and the data analysis steps were published elsewhere [21].

A water loop system was utilised to remove air from the pipes and the CFF samples prior to starting the measurements. Water at a temperature of 283 ± 3 K served as the working medium throughout the experiments. The water density (*ρ*) and dynamic viscosity (*µ*) were determined by substituting the measured temperatures into Equations (6) and (7), respectively [22,23]:

(6) $$\rho_{T}=999.842594+6.793952\times 10^{-2}T-9.095290\times 10^{-3}T^{2}+\;1.001685\times 10^{-4}T^{3}-1.120083\times 10^{-6}T^{4}+6.536332\times 10^{-9}T^{5}$$

(7) $$\log\mu =A+\frac{B}{C-T}$$

where T is the temperature (K), and the constants *A*, *B* and *C*, with values of −4.5318, −220.57 and 149.39, respectively, were employed for the temperature range of interest (270–380 K).

Finally, the permeability of the CFF samples was determined using a polynomial interpolation equation of the form f(x)=Ax, which represents the pressure gradient (Pa/m). *A* denotes the dynamic viscosity of water over the intrinsic permeability (kg/m^3^·s), and x corresponds to the superficial velocity (m/s).

## 3. Results and Discussion

### 3.1. Porosity

By assuming a homogenous structure within the CFFs, the areal porosity (*ϕ_areal_*) of the CFFs was determined by evaluating the ratio of the voids’ area to the total area, according to Equation (8) [24]. The area of the voids was obtained through surface integration over the geometrical pattern, while the total area was calculated based on the CFF sample dimensions.

(8) $$\emptyset_{areal}=\frac{A_{v}}{A_{t}}$$

To validate the porosity obtained from the numerical approach, the total porosity of the CFFs of grades 30 and 50 was estimated experimentally using Equation (9). This equation represents the fraction of the volume of the voids (*V_voids_*) over the total volume (*V_total_*). The total porosity was determined using the density of the CFF ($\rho_{CFF}$) obtained from the measured mass and volume of the CFFs, as well as the particle density ($\rho_{P}$) measured using a gas displacement pycnometer system (AccuPyc II 1340).

(9) $$\phi =\frac{V_{v}}{V_{b}}=\frac{\left(V_{b}-V_{s}\right)}{V_{b}}$$

Table 5 summarises the porosity values obtained from the numerical CFD model and the experimental measurements. A good correlation, with less than 2% deviation, can be observed between the experimental and the modelling results. The higher porosity of the CFFs of grade 30 obtained from the model may be attributed to the breaking of the filter *Struts* while cutting the CFFs, which reduced the number of channels. On the other hand, the slightly lower porosity of the CFFs of grade 50 obtained from the model compared to the experimental result was due to the slight non-homogeneity of the prepared geometric pattern, particularly in the middle section, as shown in Figure 3. This non-homogeneity led to a slight reduction in the area of the voids.

### 3.2. Velocity Fields

The velocity fields within the channels of the 2D geometric patterns of the CFFs were modelled by solving the Stocks equation in the creeping flow interface. Figure 4a,b present the velocity magnitude inside the channels of the CFFs of grades 30 and 50, respectively, using a colour map. The streamlines and velocity vectors represent the instantaneous direction of the fluid motion and the velocity magnitude, respectively. The colour contours indicate the local velocity magnitude, while the velocity vectors demonstrate that the interstitial velocity of the CFFs of grade 50 was higher than that of the grade 30 CFFs for approximately equal superficial velocities (≈1 mm/s).

**Figure 5:**
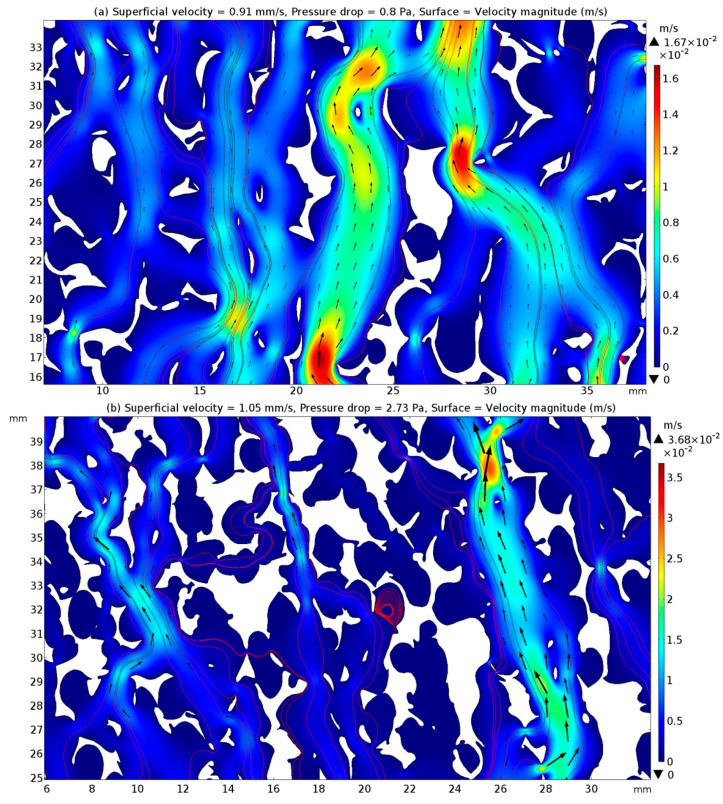
Colour map showing the velocity magnitude within the channels of the 2D geometrical patterns of the CFFs of (**a**) grade 30 and (**b**) grade 50.

In Figure 5a,b, the width of some channels appears larger than the experimentally measured mean pore diameter of the CFFs, which can be attributed to the fragmentation of the CFF filaments during the cutting process.

The average interstitial velocity over the 2D domain of the geometric pattern was evaluated during post-processing and compared to the intrinsic velocity ($\bar{v}$) calculated using the Dupuit–Forchheimer equation (see Equation (10) below). The Dupuit–Forchheimer equation relates the average intrinsic velocity to the volumetric flux, i.e., the superficial velocity and the porosity [13]:

(10) $$\bar{v}=\frac{J\;\left(volumetric\;flux\right)}{\phi \;\left(\mathrm{Porosity}\right)}$$

**Figure 6:**
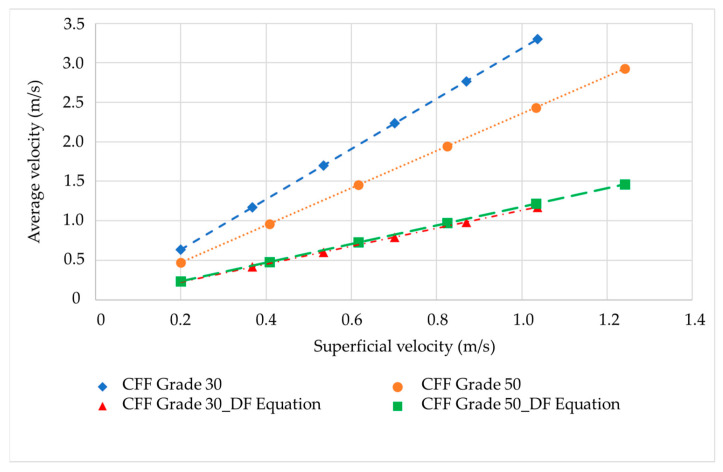
Average intrinsic velocity as a function of the superficial velocity obtained from the numerical CFD model and the Dupuit–Forchheimer equation.

Figure 6 illustrates the correlation between the intrinsic velocity obtained from the numerical CFD model and the Dupuit–Forchheimer equation for values of superficial velocity ranging from approximately 0.2 to 1 mm/s for the CFFs of grades 30 and 50. The intrinsic average velocity obtained from the numerical CFD model proved to be higher than that obtained with the Dupuit–Forchheimer equation. The Dupuit–Forchheimer equation yielded equal average intrinsic velocities for the CFFs of grades 30 and 50, as the porosity is identical for both grades. However, the numerical CFD model indicated a higher average intrinsic velocity for the CFFs of grade 30 compared to the CFFs of grade 50. The lower average intrinsic velocity of the CFFs of grade 50 can be attributed to some disconnected channels, i.e., to dead zones, within the geometric pattern, which exhibits a nearly zero velocity magnitude.

### 3.3. Permeability

The permeability of the CFFs of grades 30 and 50 was determined during post-processing using the user-defined variable *k*, see Table 2, and the results are summarised in Table 6 along with the mean permeability of the corresponding CFFs obtained from the pressure drop experiments. The numerical CFD models yielded higher permeability values for the CFFs of both grade 30 and grade 50. However, the relative difference between the permeability obtained from the numerical CFD model and that determined with the experimental measurements was larger for the CFFs of grade 30 compared to those of grade 50 (by approximately one order of magnitude). This difference was mainly due to a more significant disparity between the modelled and the experimentally obtained superficial velocity for the CFFs of grade 30.

## 4. Conclusions

A novel method was employed to generate a 2D macro-structural model of CFFs for the CFD modelling of their hydraulic properties. The geomaterial pattern of the CFFs was imported into COMSOL Multiphysics^®^ to simulate a fluid flow through the filters using the creeping flow interface. This simulation was performed within a flow velocity range where the *Re_i_*, calculated based on experimentally determined superficial velocity values, was less than 1. The analysis yielded the following conclusion:

1. The introduced procedure successfully generated a 2D geometrical pattern of CFFs that accurately replicated the tortuous surface path through the CFFs, for which fluid flow could be simulated.
2. The areal porosity obtained through 2D CFD modelling was comparable to the experimentally determined porosity.
3. The CFD modelling revealed that the interstitial velocity, obtained by averaging the instantaneous velocity throughout the macro-structure, was higher than the intrinsic velocity calculated using the Dupuit–Forchheimer equation, which relies on the superficial velocity and porosity of the porous medium, i.e., of the CFF.
4. The permeabilities obtained from the CFD modelling using COMSOL Multiphysics^®^ were higher than the experimentally obtained permeabilities. This discrepancy was attributed to the non-representative nature of the geometrical pattern employed for the investigated CFFs.

The present study’s overall findings emphasise the capability of the introduced methodology to generate an accurate numerical 2D representation of CFFs, enabling the simulation of a fluid flow through the filters. However, the observed differences in interstitial velocity and permeability highlight the need for further refinement and optimisation of the geometrical pattern to improve the representativeness of the model.

## 5. Future Work

Preparing reproducible CFF samples poses significant challenges, demanding a more accurate and reliable approach. An advanced method involving impregnating CFF samples into castable metallographic resins prior to sectioning will be employed to support and reinforce the CFF *Struts* during the cutting process, thus preventing their fragmentation and preserving the samples’ integrity.

The effect of differing meshing methods will also be explored to optimise the sample geometry to better suit the requirements of the numerical 2D macro-structural CFF simulation model. This will, in turn, contribute to securing the best possible sample configuration for accurate analysis and performance evaluation of CFFs.

## Figures and Tables

**Table 1:** Defined parameters for the numerical CFD model, their values, units, and descriptions, G stands for grade.

Name	Expression	Value	Description
**rho0**	999.7 kg/m^3^	999.7 kg/m^3^	Fluid density
**eta0**	0.0013 kg/m·s	0.0013 kg/m·s	Fluid dynamic viscosity
**p0**	0.17313892 Pa	0.17314 Pa	Pressure (inlet)
**H (CFF G30)**	50 mm	0.05 m	Domain height
**L (CFF G30)**	44.724 mm	0.044724 m	Domain thickness
**H (CFF G50)**	50 mm	0.05 m	Domain height
**L (CFF G50)**	46 mm	0.046 m	Domain thickness

**Table 2:** User-defined variables for postprocessing evaluation of the permeability.

Name	Expression	Unit	Description
***q***	(comp1.spf.out1.Mflow) */rho0	m^3^/s	Volumetric flow rate
***u***	q/H/1 m	m/s	Superficial velocity
***k***	u × eta0 × H/p0	m^2^	Permeability

* (comp1.spf.out1.Mflow) is the mass flow (kg/s), which is defined as a variable under the physics node.

**Table 3:** Boundary conditions used during the analyses.

Boundary Type	Boundary Condition and Value
**Inlet**	Pressure, *P* = *P0*
**Outlet**	Pressure, *P* = 0
**Conduits walls**	Wall, no slip
**Outer walls**	Wall, no slip

**Table 4:** Assumptions made in the analyses.

Assumption	Description
Stationary study	Variables are not changing with time
*ρ* = constant	Incompressible fluid
*G* = 0	Gravitational force is zero
*T* = constant	Isothermal fluid

**Table 5:** Porosity values obtained from the numerical CFD model and experimental measurements for the CFFs of grades 30 and 50.

CFF Grade	Numerical CFD Model	Experimental
**30**	89.43%	88.5 ± 0.58%
**50**	83.16%	84.7 ± 0.48%

**Table 6:** The numerically and experimentally determined permeability of the CFFs of grades 30 and 50.

CFF Grade	Permeability (Experiment)	Permeability (CFD Modelling)
30	6.28·10^−8^ m^2^	1.72·10^−7^ m^2^
50	2.45·10^−8^ m^2^	3.90·10^−8^ m^2^

## Data Availability

All the data created are presented in the paper.

## Nomenclature

Symbol	Unit	Description
*ρ*	Kg/m^3^	Density
*u*	m/s^1^	Superficial velocity
*m*	kg	Mass
*V_p_*	m^3^	Pore volume
*ϕ*	dimensionless	Porosity
*V_t_*	m^3^	Total volume
*J*	m/s^1^	Volumetric flux
Δ*P*	Pa	Pressure drop
∇*P*	kg/m^2^∙s^2^	Pressure gradient
*µ*	Kg/m^1^·s^1^	Dynamic viscosity
*L*	m	Domain thickness
*H*	m	Domain height
*Q*	m^3^/s^1^	Volumetric flow rate
*k*	m^2^	Permeability
*u*	m/s^1^	Velocity vector
*β*	1/m^1^	Inertia resistance factor
*ϕ_areal_*	dimensionless	Areal porosity
*A_v_*	m^2^	Area of voids
*A_t_*	m^2^	Total area
*d_p_*	m	Spherical particle diameter
*Re*	Dimensionless	Reynolds number
*d_C_*	m	Cell’s Feret diameter
*d_W_*	m	Window’s Feret diameter
*d_S_*	m	Strut’s length
*V_v_*	m^3^	Volume of voids
*V_b_*	m^3^	Volume of bulk
*V_s_*	m^3^	Volume of solid matrix
*T*	°C	Temperature
*P*	Pa	Pressure
*F*	-	All forces other than pressure
∇^2^	-	Laplace operator
*D_ρ/_D_t_*	-	Substantial time derivative
$\bar{v}$	m/s^1^	Intrinsic average velocity
*rho0*	Kg/m^3^	Fluid density
*eta0*	Kg/m^1^·s^1^	Fluid dynamic viscosity
*p0*	Pa	Pressure (inlet)
